# Sarcopenia and quality of life in patients with diabetes mellitus type 2 in a maternal and child center

**DOI:** 10.3389/fnut.2025.1657162

**Published:** 2025-12-05

**Authors:** José Luis Guzmán Mallqui

**Affiliations:** Doctorado en Nutrición y Alimentos, Escuela de Posgrado, Universidad San Ignacio De Loyola, Lima, Peru

**Keywords:** sarcopenia, quality of life, diabetes mellitus, handgrip strength, EWGSOP2

## Abstract

**Objective:**

To determine the relationship between sarcopenia and quality of life in patients with type 2 diabetes mellitus in a mother and child center, Peru 2024.

**Methods:**

The research was applied, non-experimental, relational, where work was done in 295 diabetic patients. Regarding the technique, observation was used, recording data on a data collection form, and the survey technique was applied, where the SF-36 questionnaire was administered. Grip strength was measured using the Jamar hydraulic dynamometer and the measurement method according to the European Working Group on Sarcopenia in Older People 2 (EWGSOP2).

**Results:**

Normal grip strength was most represented at 57.29%, followed by low grip strength at 42.71% using the EGWSOP criterion. On the other hand, according to the JAMAR method, low grip strength was 61.69%. Regarding quality of life, the average level was obtained (27.12%), high (26.10%), followed by the very high level (18.98%). In addition, a significant correlation was determined between the dimensions of physical function, physical role, bodily pain perception, general health, vitality, social function, emotional role, and mental health of quality of life and sarcopenia (*p* < 0.001).

**Conclusion:**

It is concluded that there was a positive and significant correlation between sarcopenia and quality of life in patients with type 2 diabetes mellitus for both the EGWSOP criterion (r = 0.303) and the JAMAR method (r = 0.306) (*p* < 0.001).

## Introduction

Sarcopenia is a condition characterized by a progressive loss of muscle mass, strength, and physical function that progresses with advancing age (1). This condition is characterized by a range of adverse events, including physical disability, impaired quality of life, and increased mortality (2). This condition is often assessed by physical measurements of muscle mass and size (3, 4). Alternatively, physical performance may be assessed as an indicator of sarcopenia by tests such as handgrip strength, lower extremity strength, walking speed, or various functional routines (4).

A progressive decline in muscle mass occurs at an annual rate of 1 to 2% after age 30 and accelerates to 1.5 to 3% per year after age 60. Although muscle atrophy occurs with aging, the fact that there is variability in this process indicates that there are potential factors that could influence the rate of decline in muscle strength and function (5). With aging, significant physiological changes occur that may be associated with a decrease in muscle tissue and the development of sarcopenia, this being the result of an imbalance between the anabolic and catabolic pathways that control muscle mass; having as causes inadequate nutrition, especially poor protein intake, reduced physical exercise, among others (6, 7).

At the international level, findings from the Asian Working Group for Sarcopenia (AWGS) showed a prevalence of 15% in Chinese (>60 years) and Japanese (≥65 years) adults with type 2 diabetes mellitus (T2DM), demonstrating in the study that diabetes and sarcopenia interact with each other (8). Another study conducted in Greece showed that patients with type II diabetes mellitus had a 55% higher risk of sarcopenia compared to those without diabetes (9).

At the national level, a study developed in Lima showed that the prevalence of probable sarcopenia defined as weak grip strength was 46.5%, and was independently associated with probable sarcopenia in adults who were hospitalized in a national hospital (10).

Skeletal muscle accounts for 40% of total body weight and is an important part of the locomotor system, as almost all body activities are controlled by its contraction. Since muscle is the primary site of glucose uptake, reduced muscle mass leads to increased insulin resistance. Lipid accumulation within muscle tissue and reduced mitochondrial function are other factors that lead to decreased insulin sensitivity (11).

Insulin stimulates protein synthesis, including muscle protein synthesis, and defects in its production can lead to reduced muscle protein synthesis and increased protein breakdown leading to sarcopenia (12). Therefore, persistent hyperglycemia increases the production of advanced glycation end products (AGEs) that accumulate in muscle and cartilage causing muscle stiffness and reduced muscle function (13, 14).

The European Society of Clinical Nutrition and Metabolism and the European Association for the Study of Obesity (ESPEN-EASO) recommend handgrip strength (HGS) and the chair rise test (CST) to assess muscle strength, with the CST being a convenient surrogate for lower limb strength (15).

The quality of life of patients with type 2 diabetes mellitus can be affected in a variety of ways, including physical, emotional, and social aspects (16, 17). Therefore, maintaining blood glucose levels within normal ranges is crucial to prevent complications; however, this may require significant dietary changes, regular physical activity, and strict adherence to medication, which can be challenging and stressful (18, 19).

Therefore, this article aims to explore the relationship between sarcopenia and quality of life based on the study of patients with type II diabetes mellitus.

## Materials and methods

This research is characterized by being applied, non-experimental, relational, cross-sectional and prospective (20) to analyze sarcopenia and quality of life in patients diagnosed with type II diabetes mellitus at the Juan Pablo II Maternal and Child Health Center, an establishment that has category I-3, located in the district of Villa el Salvador, city of Lima - Peru, which has the services of Growth and Development, immunizations, nutrition, pediatrics gynecology, general medicine, tuberculosis program, delivery room and emergency. The sample included 295 patients over 18 years of age, diagnosed with type II diabetes mellitus who regularly attended the maternal and child center, which were chosen through simple random sampling.

To assess sarcopenia, grip strength was measured using a Jamar Hydraulic dynamometer, recognized as the gold standard in dynamometry. It is compact and portable, although it weighs relatively little at 680 grams. Its dial displays force in kilograms and pounds, with markings in 2-kg or 5-pound intervals. It requires between 1.4 and 1.8 kg of force to move the indicator needle and has a greater margin of error at lower loads. The dynamometer has a handle that can be adjusted to five different positions (2.5, 3.8, 5.1, 6.4, and 7.6 cm apart), so it has been observed that maximum grip strength is generally achieved in position II or III of the device. To evaluate the amount of strength, the measurements agreed upon by the European Working Group on Sarcopenia in Older People 2 (EWGSOP2) (21) were taken into account, where the following cuts are taken into account: Grip strength <27 kg for men and <16 kg for women.

There are various protocols for measuring handgrip strength, but to standardize the procedure and compare results with other studies, the technique recommended by the American Society of Hand Therapists (ASHT) was followed. This technique suggests that the participant be seated, with the shoulder adducted and not rotated, the elbow flexed at 90° and close to the body. The forearm and wrist should be in a neutral position, without using armrests. The participant held the dynamometer handle with a cylindrical grip, so that the indicator was visible to the evaluator. Each participant performed three maximum attempts for each measurement. The results of the three attempts were recorded for both hands, dominant and non-dominant, with a one-minute rest recommended between each attempt, for a total of 7 min for each measurement. An average of 20 patients were evaluated per day.

In relation to the established indicators, the following definitions of BMI were worked on: Underweight (less than 18.5 kg/m^2^), Normal (18.5–24.9 kg/m^2^), Overweight (25–29.9 kg/m^2^) and Obesity (30 kg/m^2^ or more) (22).

Regarding quality of life, the SF-36 questionnaire was used, which comes from the study by Vilagut et al. (23) in its original Spanish version. It includes 36 items, each with response options that vary according to the item according to a Likert scale, being an instrument widely used and validated in multiple international studies. Likewise, a reliability test was performed on a sample of 20 patients, where according to the Cronbach’s alpha test, a coefficient of 0.818 was obtained, demonstrating a high level of reliability. Regarding the score of this questionnaire, 0 represents the worst health state and 100 the best possible state, where the number ranges from two to six alternatives depending on the item. The scores are calculated for each of the eight dimensions which are physical function, physical role, bodily pain, general health, vitality, social function, emotional role and mental health, classifying quality of life as good, fair and poor.

The SF36 questionnaire was then administered to each participant in a sealed envelope. Participants had a maximum of 20 min to complete all items in an orderly manner. The questionnaires were distributed in a manilla envelope, and each participant was given a pen. Upon completion, the envelope was sealed and stored.

Regarding the statistical analysis, the Kolmogorov-Smirnoff normality test was performed, where the data were normally distributed. Therefore, the Pearson correlation test was applied (see Table 1).

**Table 1:** Description of the general characteristics of the patients.

Characteristics	*N* (295)	%
Sex		
Male	71	24.07
Female	224	75.93
Age		
From 18 to 30 years old	34	11.53
From 31 to 65 years old	163	55.25
From 66 years old and over	98	33.22
BMI		
Thinness	23	7.80
Thinness 1	4	1.36
Normal	81	27.46
Obesity	21	7.12
Obesity 1	47	15.93
Obesity 2	13	4.41
Obesity 3	11	3.73
Overweight	95	32.20
Weight (mean ± SD)	69.42 ± 16.98	
Height (mean ± SD)	1.56 ± 0.08	

Source: researcher’s own.

## Results

The sample analyzed in this study included 295 subjects diagnosed with type 2 diabetes mellitus. Females predominated (75.93%), while men accounted for 24.07%. The majority were aged 31–65 years (55.25%), followed by those aged 66 and over (33.22%), and young adults aged 18–30 years (11.53%). Regarding nutritional status, 32.20% were overweight, and 27.46% had a BMI within the normal range. Different degrees of obesity were identified: type I (15.93%), type II (4.41%), and type III (3.73%). Likewise, 7.80% were thin and 1.36% were type I thin. Regarding weight, the average was 69.42 ± 16.98 kg and the mean height was 1.56 ± 0.08 m (see Table 2).

**Table 2:** Baseline demographic characteristics.

Demographic characteristics	*n*	%
Type of housing		
Own house	172	58.3
Rented	83	28.1
Loaned/family	40	13.6
Predominant material		
Brick/cement	229	77.6
Wood or others	66	22.4
Basic services		
Drinking water	246	83.4
Drain	231	78.3
Electricity	282	95.6
Internet	119	40.3
Number of people at home		
1–3 people	104	35.3
4–6 people	143	48.5
≥ 7 people	48	16.2

Data are presented in absolute frequencies (*n*) and percentages (%).

The majority of participants lived in their own homes (58.3%), while just over a quarter lived in rented accommodation (28.1%), and a smaller group lived in homes provided by relatives (13.6%). Regarding the predominant material, the vast majority lived in brick or cement buildings (77.6%), while 22.4% lived in homes made of wood or other materials, reflecting a certain heterogeneity in housing conditions.

Regarding access to basic services, coverage was high for electricity (95.6%) and drinking water (83.4%), although internet access was relatively low (40.3%), which could imply limitations in communication and virtual education. Finally, regarding household composition, almost half of the participants lived in families of 4 to 6 members (48.5%), followed by small households of 1 to 3 people (35.3%), while 16.2% belonged to larger households (≥7 members), which could have implications for family dynamics and the distribution of resources within the household (see Table 3).

**Table 3:** Weight distribution in relation to the three age groups.

Age group (years)	Underweight f(%)	Normal weight f(%)	Overweight f(%)	Obesity f(%)	Total
18–30 years old	2 (5.0%)	18 (45.0%)	12 (30.0%)	8 (20.0%)	40
31–65 years old	5 (2.8%)	45 (25.0%)	72 (40.0%)	58 (32.2%)	180
≥ 66 years	2 (2.7%)	14 (18.7%)	28 (37.3%)	31 (41.3%)	75
Total	9 (3.1%)	77 (26.1%)	112 (38.0%)	97 (32.9%)	295

Weight classification was made according to the Body Mass Index (BMI), according to the World Health Organization.

Weight distribution by age group showed that only 3.1% of participants were underweight, while 26.1% maintained a normal weight. The largest proportion was concentrated in the overweight (38.0%) and obese (32.9%) categories, reflecting that more than two-thirds of the sample were overweight. Normal weight predominated among young adults (18–30 years old) (45.0%), while the prevalence of overweight (40.0%) and obesity (32.2%) increased among adults aged 31–65 years. Obesity reached its highest level (41.3%), with 37.3% being overweight, indicating that more than 78% of this group was overweight. These results reflect a growing trend toward overweight and obesity with advancing age (see Table 4).

**Table 4:** Characterization of sarcopenia.

Sarcopenia	*N* (295)	%
Diagnosis according to EGWSOP		
Low grip strength	126	42.71
Normal grip strength	169	57.29
Diagnosis according to JAMAR		
Normal	113	38.31
Decreased	182	61.69

Sarcopenia was assessed using the European Working Group on Sarcopenia in Older People (EGWSOP) criteria and manual dynamometry (JAMAR).

The results of both methods show a high prevalence of muscle weakness in the sample, with the first method showing slightly less than half having low grip strength, while with the second method, more than half having decreased strength (see Table 5).

**Table 5:** Frequency distribution of the level of quality of life with its dimensions.

Dimensions	Levels or ranks									
	Very low		Low		Half		High		Very high	
	*n*	%	*n*	%	*n*	%	*n*	%	*n*	%
Physical function	43	14.58	77	26.10	104	35.25	51	17.29	20	6.78
Physical role	38	12.88	27	9.15	37	12.54	67	22.71	126	42.71
Body pain	129	43.73	25	8.47	24	8.14	13	4.41	104	35.25
General health	111	37.63	23	7.80	0	0.00	39	13.22	122	41.36
Vitality	11	3.73	25	8.47	69	23.39	99	33.56	91	30.85
Social function	37	12.54	45	15.25	79	26.78	63	21.36	71	24.07
Emotional role	37	12.54	96	32.54	122	41.36	34	11.53	6	2.03
Mental health	11	3.73	49	16.61	123	41.69	87	29.49	25	8.47
Quality of life	20	6.78	62	21.02	80	27.12	77	26.10	56	18.98

Quality of life levels were categorized into five ranges in relation to the score corresponding to the SF-36 questionnaire.

When analyzing the overall level of quality of life, the majority of patients were concentrated in the medium (27.12%) and high (26.10%) levels, followed by the very high level (18.98%). However, 21.02% were in the low level and 6.78% in the very low level, indicating that while the majority had a moderate to favorable perception of their quality of life, significant gaps remained to be addressed.

Pearson correlation analysis showed positive and statistically significant associations between sarcopenia and different dimensions of quality of life in patients with type 2 diabetes (Table 6). Overall, sarcopenia was related to total quality of life (EGWSOP r = 0.303; JAMAR r = 0.306; *p* < 0.001). Regarding specific dimensions, low magnitude correlations were observed with physical function (r = 0.202 and r = 0.201), physical role (r = 0.172 and r = 0.168), general health (r = 0.155 and r = 0.150), and mental health (r = 0.186 and r = 0.189), all with statistical significance (*p* ≤ 0.01). Likewise, slightly higher magnitude correlations were found with bodily pain (r = 0.276 and r = 0.284), vitality (r = 0.238 and r = 0.242), emotional role (r = 0.233 and r = 0.236) and especially with social function (r = 0.334 and r = 0.347), the latter being the most intense within the dimensions evaluated (*p* < 0.001). These findings indicate that greater muscular involvement is associated with a progressive deterioration in different spheres of quality of life, with a more marked impact on the social interaction of patients.

**Table 6:** Relationship between sarcopenia and overall quality of life and its dimensions.

Dimension of quality of life	Sarcopenia according to EGWSOP (r)	Sig. (bilateral)	Sarcopenia according to JAMAR (r)	Sig. (bilateral)
Global quality of life	0.303	0.000	0.306	0.000
Physical function	0.202	0.000	0.201	0.000
Physical role	0.172	0.003	0.168	0.003
Body pain	0.276	0.000	0.284	0.000
General health	0.155	0.007	0.150	0.009
Vitality	0.238	0.000	0.242	0.000
Social function	0.334	0.000	0.347	0.000
Emotional role	0.233	0.000	0.236	0.000
Mental health	0.186	0.001	0.189	0.001

r values correspond to Pearson’s correlation coefficient. All analyses were performed with a significance level of *p* < 0.05.

## Discussion

The present study was conducted in 295 participants diagnosed with type II diabetes. Sarcopenia was assessed using the JAMAR method using a dynamometer and the protocol established by the EGWSOP. Quality of life was also evaluated using questionnaires that collected information on this variable. These findings present similarities and differences in relation to the variables evaluated, which will be presented.

Likewise, the corrected arm muscle area (AMB) revealed that 50.17% of patients had decreased muscle mass, and 11.86% were at risk of low muscle mass. Only one third of patients (31.53%) showed muscle mass within normal ranges, and less than 7% had good or increased levels. On the other hand, it was determined that normal grip strength was most represented with 57.29% followed by low grip strength with 42.71% using the EGWSOP criteria. On the other hand, according to the JAMAR method, low grip strength was 61.69% demonstrating that there is a high prevalence of muscle weakness in patients with type 2 diabetes mellitus. These results are related to those found by Du et al. (8), where the overall prevalence of sarcopenia was 30.2%; 41.3% in men and 20.1% in women. Furthermore, a low body mass index (BMI), testosterone concentration (for men), and muscle reserve assessment were found to be predictive significance for sarcopenia in T2DM (*p* < 0.05). This may be explained by the close relationship between type 2 diabetes mellitus and the progressive loss of muscle mass and strength. Factors such as a low body mass index and reduced muscle mass can also directly influence grip strength. Therefore, both results coincide in pointing to a functional impairment related to the loss of muscle strength, which is one of the central components of sarcopenia in this type of patient.

According to the EGWSOP, approximately 60% of those with low grip strength are concentrated within the low, very low, and medium quality of life ranges. Therefore, patients with low grip strength would be associated with a poorer perception of quality of life. Meanwhile, normal grip strength is associated with a high or very high quality of life. In the analysis of quality of life and its dimensions, it was observed in the physical function dimension that the highest percentage of patients were located at a medium level (35.25%), followed by low (26.10%) and very low (14.58%). Regarding mental health, 41.69% of patients were at the medium level, while 29.49% reached a high level. Along the same lines, Zhang et al. (24) determined in terms of quality of life, that patients with diabetes had a lower score in the physical component summary (PCS) (*p* = 0.001), the mental component summary (MCS) (*p* = 0.005) and the 36-item short-form health survey (SF-36) (*p* = 0.001), determining that nutritional aspects such as grip strength and quality of life were considered low compared to people diagnosed with diabetes mellitus. This occurs because the patient with diabetes mellitus presents a progressive advancement of the disease as time goes by, so their quality of life is impaired, since many of them lose strength, muscle mass and if the disease progresses they may have degenerative mental complications, so many of these dimensions are directly related to sarcopenia in this vulnerable population.

At the study level, a positive, weak and significant relationship was found between sarcopenia and the physical role component dimension of quality of life in people with type 2 diabetes both according to the EGWSOP criteria (r = 0.172) and the JAMAR method (r = 0.168); *p* = 0.003. This is related to the study conducted by Runzer et al. (25), which found a significant relationship between weak grip strength and poor physical performance (ORa: 4.32, 95% CI: 1.97 to 9.59) as well as the performance of daily activities. These results are supported by the study by Shrestha and Gurung (26) which concludes that handgrip strength was significantly lower in subjects with type 2 diabetes mellitus compared to healthy volunteers. This occurs because chronic hyperglycemia promotes a low-grade systemic inflammatory state, which promotes muscle protein breakdown and reduces protein synthesis. Furthermore, insulin resistance, a central characteristic of this condition, interferes with glucose uptake by muscle fibers, decreasing the energy availability needed for muscle contraction and tissue regeneration. This leads to a decrease in muscle mass and strength, essential components of physical performance.

A weak but significant positive correlation was observed between sarcopenia and the emotional role dimension of quality of life in people with type 2 diabetes mellitus for both the EGWSOP criterion (r = 0.233) and the JAMAR method (r = 0.236), *p* < 0.001. The same was true for the mental health dimension, where these findings suggested that muscle involvement was associated with lower emotional and psychological well-being in patients. Likewise, Panahi et al. (27) estimated the physical (PCS) and mental (MCS) components of quality of life, where they determined that individuals with diabetes had higher PCS (40.9 ± 8.8 vs. 42.7 ± 8.6, *p*-value <<0.001) and MCS scores (45.0 ± 10.2 vs. 46.4 ± 9.4, *p*-value <0.001) compared to participants without diabetes, concluding that people with diabetes showed reduced quality of life scores. Similarly, the study conducted by Veiga et al. (28) concluded that grip strength in diabetic patients was decreased. In addition, there is a significant association between grip strength and mental health. This occurs because hyperglycemia increases the risk of neurovascular inflammation, affecting areas of the brain related to memory, mood, and emotional regulation. Furthermore, diabetes has been shown to alter the flow and levels of serotonin and dopamine, resulting in neurodegenerative damage.

On the other hand, patients with diabetes mellitus also face constant psychosocial stress due to continuous disease management, dietary restrictions, the risk of chronic complications, and the perception of loss of autonomy, all of which can predispose or aggravate disorders such as anxiety and depression. The coexistence of sarcopenia, by limiting mobility and functional independence, can reinforce this vicious cycle, increasing the feeling of frustration, isolation, and emotional burden, which is reflected in lower scores on the emotional dimensions of quality of life. These results are supported by the study conducted by Sravya et al. (29), where sarcopenia was evaluated using the Jammer hydraulic dynamometer to measure handgrip strength, concluding that people with T2DM are prone to sarcopenia, and that this is associated with a low BMI, as well as poor physical activity.

The present study concluded that there is a positive and statistically significant correlation between sarcopenia and quality of life in patients with type 2 diabetes mellitus, observed when applying the diagnostic criteria proposed by the EWGSOP (r = 0.303) as well as the JAMAR method (r = 0.306) (*p* < 0.001). Unlike what was found by Palacios et al. (30) in which they concluded that there is no association between type 2 diabetes mellitus, muscle strength and physical performance. Therefore, it is essential to consider the limitations in terms of sample size and differences in the characteristics of the studied populations, such as age range, duration and control of diabetes, the presence of comorbidities (such as obesity, neuropathy or cardiovascular diseases), the level of physical activity or even the socioeconomic and cultural environment. All these factors can influence the degree of muscle loss, functionality and perception of quality of life.

Therefore, the main strength of this study is the selection of a problem of high clinical and epidemiological relevance. The identification of a statistically significant association between both variables supports the hypothesis that sarcopenia is a determining factor in the functional decline of diabetic patients, opening up prospects for the implementation of routine muscle function assessments in this group.

Finally, this work contributes to the expansion of scientific evidence in Latin American contexts, where studies on sarcopenia in diabetes are still scarce. The application of appropriate analytical methods strengthened the internal validity of the results, allowing for solid inferences about the relationship between the variables investigated. From a clinical perspective, the findings emphasize the need to develop systematic screening and management strategies for sarcopenia in patients with type 2 diabetes mellitus, with the goal of preserving their quality of life, self-esteem, and functional independence, and preventing future complications, in line with internationally recommended comprehensive care approaches.

To effectively support vulnerable patients, it is necessary to apply an equity approach that considers the social determinants of health described in the PROGRESS framework (31). In this regard, strategies are proposed to ensure equitable access to health services through the expansion of primary care, telemedicine and flexible hours, as well as the cultural and linguistic adaptation of information, respecting religious beliefs and providing materials in accessible formats.

## Conclusion

The study concluded that there is a positive and significant correlation between sarcopenia and quality of life in people with type 2 diabetes mellitus, as evidenced by the EGWSOP and JAMAR criteria. This relationship varied in intensity depending on the SF-36 dimensions evaluated. A higher prevalence of muscle weakness was observed, especially when applying the JAMAR method, and a mostly moderate to favorable perception of quality of life in the participants. The most relevant correlations were found with the dimensions of social function and perception of bodily pain, followed by vitality, physical function, emotional role, general health, physical role, and mental health, all with statistical significance, indicating that the presence of sarcopenia impacts various aspects of well-being in this population.

## Data Availability

The raw data supporting the conclusions of this article will be made available by the authors, without undue reservation.
